# Community Health Workers and Pandemic Preparedness: Current and Prospective Roles

**DOI:** 10.3389/fpubh.2019.00062

**Published:** 2019-03-26

**Authors:** Matthew R. Boyce, Rebecca Katz

**Affiliations:** Center for Global Health Science & Security, Georgetown University, Washington, DC, United States

**Keywords:** community health worker, Ebola, health security, pandemic, preparedness, resilience, Zika

## Abstract

Despite the importance of community health workers (CHWs) to health systems in resource-constrained environments, relatively little has been written about their contributions to pandemic preparedness. In this perspective piece, we draw from the response to the 2014 Ebola and 2015 Zika epidemics to review examples whereby CHWs contributed to health security and pandemic preparedness. CHWs promoted pandemic preparedness prior to the epidemics by increasing the access to health services and products within communities, communicating health concepts in a culturally appropriate fashion, and reducing the burdens felt by formal healthcare systems. During the epidemics, CHWs promoted pandemic preparedness by acting as community-level educators and mobilizers, contributing to surveillance systems, and filling health service gaps. Acknowledging the success CHWs have had in these roles and in previous interventions, we propose that the cadre may be better engaged in pandemic preparedness in the future. Some practical strategies for achieving this include training and using CHWs to communicate One Health information to at-risk communities prior to outbreaks, pooling them into a reserve health corps to be used during public health emergencies, and formalizing agreements and strategies to promote the early engagement of CHWs in response actions. Recognizing that CHWs already play a role in pandemic preparedness, we feel that expanding the roles and responsibilities of CHWs represents a practical means of improving pandemic and community-level resilience.

## Introduction

Disasters are unexpected events that imply serious health, economic, or political threats, and require special considerations beyond routine procedures or resources. Large-scale infectious disease outbreaks, therefore, represent one manifestation of such events. Importantly, these disease outbreaks appear to be increasing in frequency (1), and this year alone, there have been outbreaks of cholera, Ebola, Lassa fever, Middle East respiratory syndrome, Nipah, Rift Valley fever, and yellow fever (2).

Promoting disaster resilience represents one approach to mitigate the consequences of disasters. The Sendai Framework for Disaster Risk Reduction describes disaster resilience as the ability of an entity to resist, acclimate to, and recover from the effects of a hazard, including through the preservation and restoration of essential structures and functions. Disaster resilience theory further categorizes resilience as being either inherent or adaptive (3), with inherent resilience referring to the conditions, characteristics, and properties associated with absorptive capacity; and adaptive resilience involving the activation of resources and blending preplanned and reactive actions in response to disaster-related demands.

The International Health Regulations 2005 (IHR) and the corresponding Joint External Evaluation Tool (JEE) are the preeminent international frameworks for building and assessing resilient public health systems. These assigned new responsibilities to the World Health Organization (WHO) and nations to share resources, information, and expertise to build capacities to help prepare the world for preventing, detecting, and responding to health emergencies. To this end, the JEE tool states that countries should have a skilled and competent workforce of physicians, veterinarians, biostatisticians, laboratory scientists, livestock professionals, and field epidemiologists for maintaining sustainable public health surveillance and response mechanisms.

Still, this guidance prescribes a national-level workforce and assumes that capacity will be dispersed throughout a country. This assumption is rarely a reality, particularly in low-resource or unstable regions where healthcare workforces may be concentrated in wealthier areas of a country. Although the recently released JEE 2.0 places greater emphasis on building sub-national level capacities (4), the impacts of this reality are acutely felt at the community-level during public health crises where health systems assets can be limited or nonexistent.

Community health workers (CHWs)—lay persons trained to assist in the communication or provision of health services—represent one method for extending health services at sub-national levels, particularly in underserved or remote populations (5). CHWs are commonly trained in the context of health interventions to carry out defined functions related to healthcare delivery, but rarely have formal professional or paraprofessional certifications, or degreed tertiary education (6).

CHWs have traditionally been used to improve community health initiatives, manage the risk of infectious diseases (e.g., malaria, pneumonia, and tuberculosis), and fill gaps in healthcare systems. Accordingly, much attention has focused on their importance to primary healthcare systems. However, despite their establishment at the community level, CHWs are often under-utilized in the response to infectious disease outbreaks (7) and additional roles for CHWs in promoting pandemic preparedness exist. The WHO has released training materials for preparing CHWs to respond to respiratory disease outbreaks (8, 9) but relatively little attention has been given to their potential importance in contributing to the prevention and control of other large-scale infectious disease outbreaks. Despite this, CHWs have been involved in the response to these events. At least 950 CHWs were involved in Liberia's response to the Ebola outbreak (10) and over 1,500 CHW were involved in Côte d'Ivoire's precautionary response to the Ebola outbreak in neighboring Guinea and Liberia (11).

Acknowledging the importance of CHWs in extending health services to vulnerable populations filling health system gaps, as well as their involvement in previous outbreaks, herein we discuss several roles CHWs currently play in promoting inherent and adaptive resilience and discuss future opportunities for CHWs to better sub-national pandemic preparedness and response.

## Community Health Worker Roles in Inherent Resilience

From a public health perspective, inherent resilience could include adequate nutrition, access to clean water, effective sanitation systems, robust health systems, and other means buffering populations against public health emergencies. Accordingly, perhaps the most discernible role of CHWs in pandemic preparedness is inherent to the position—one of increasing the access to health interventions and services within communities (Box 1). These efforts can reduce the risk of many morbidities and overall mortality (12) and improve the underlying population health, in theory, reducing the susceptibility of the population to infectious disease threats.

Box 1 **Selected community health worker roles that promote inherent resilience.**

- Increase the access to health services and products within communities to improve population health and reduce the likelihood of an outbreak
- Communicate important public health concepts in a culturally appropriate fashion
- Reduce the burden felt by formal healthcare systems and improve the quality of clinical care

Another role CHWs currently fill in promoting inherent resilience is the distribution of culturally appropriate health information and supplies. In the midst of the 2015 Zika outbreak, CHWs known as “*brigadistas”* were used to communicate important information regarding Zika to reduce the risk of infection in at-risk communities prior to the peak of mosquito season (13). This proactive strategy improved community awareness about the importance of eliminating mosquito breeding sites and promoted condom use to reduce sexual transmission of the virus. Brigadistas were also used to distribute Zika prevention kits—containing barrel covers, bed nest, condoms, and educational materials—to pregnant mothers.

Finally, CHWs can act as a community-level triage system—treating those with minor illness and referring those with more serious disease. This reduces pressures on often over-burdened health systems and helps to ensure that formal healthcare cadres—those referenced in the IHR and JEE Tool—are available to provide health services to those most in need. For example, in Brazil, CHWs have been used to triage conditions and provide basic primary care to families to resolve minor ailments (14). In the event of severe issues, they were trained to refer patients to nurses or physicians who were better equipped to manage illness. Ultimately, this strategy reduced the number of hospitalizations and led to significant improvements in clinical outcomes—both with regard to mortality and improving access to healthcare.

## Community Health Worker Roles in Adaptive Resilience

With regard to pandemic preparedness, the adaptive resilience roles of CHWs are more complicated. Although CHWs receive healthcare-related training, expecting or obligating them to medically respond to a large-scale infectious disease outbreak is unethical and impractical. The disproportionate effects of outbreaks on healthcare workers—as seen in the 2003 SARS (15) and 2014 Ebola outbreaks (16)—are likely to rapidly decimate the healthcare workforce resulting in a reluctance to work and in high rates of absenteeism (17). CHWs are not exempt from this trend (18).

Still, medical tasks are fundamentally different from other essential response tasks—the former require technical proficiencies, whereas the latter can rely on social competencies, communication skills, and local-level knowledge (19). It is in these non-medical roles that CHWs can excel in contributing to the adaptive resilience of health systems (Box 2).

Box 2 **Selected community health worker roles that promote adaptive resilience.**

- Act as community-level educators, organizers, and mobilizers during infectious disease outbreaks
- Contribute to syndromic disease surveillance systems while completing routine activities
- Complete medical tasks unrelated to the infectious disease outbreak to fill health service gaps during or following the outbreak.

Community health workers often represent a trusted voice in the community and thus also represent valuable assets for social mobilization and the distribution of health information during outbreaks. A key lesson from the 2014 Ebola outbreak response was that engaging communities to contain the spread of disease can be challenging unless there was an existing network of healthcare workers embedded within communities (20, 21). Because CHWs reside in or near the communities they service, they are uniquely positioned to act as community-level educators, organizers, and mobilizers in this network. Indeed, during the 2014 Ebola response, engaging CHWs in response procedures improved the efficacy of response activities (7).

Another adaptive role that CHWs contribute to is disease surveillance. Depending on the locale from which an outbreak occurs, CHWs could be on the frontlines of responding to a public health emergency and having systems in place for CHWs to report unusual symptoms or epidemiological patterns while performing their routine activities could enhance syndromic surveillance. This role was validated in Côte d'Ivoire (11) and Sierra Leone (7) during the 2014 Ebola epidemic where CHWs conducted community surveillance activities and reported suspected Ebola cases to public health authorities.

CHWs can also promote adaptive resilience by resuming their medical roles and filling health service gaps following outbreaks. This role is of great importance should healthcare workforces be depleted by an outbreak response. Scholars have noted that sustained political and financial investment in CHW programs could create a solid foundation for CHWs to close sudden or unexpected health system gaps and improve the resilience of health systems (18).

## Future Opportunities: Recommendations for Engaging Community Health Workers

Given these roles in promoting resilience, better involving CHWs in pandemic preparation efforts appears both logical and practical. There have been direct calls for sustained investment in health worker training, which could include CHWs, to mitigate the risks posed by disease outbreaks (16, 22) and considering the potential contributions of CHWs in containing public health emergencies, scaling-up CHW strategies could avoid an estimated $750 million in economic losses per year (23). We now propose several actionable options for improving CHW trainings and involvement in health emergency response to better promote pandemic preparedness.

First, since CHWs play a key role in providing health services, and often work on a voluntary basis, their personal satisfaction, and motivation are central to their involvement in health interventions. While much work has investigated CHW's motivations for routine activities (24–28), less evidence exists regarding CHWs motivations, satisfaction, and role perception when providing services in environments that warrant special considerations (e.g., during an outbreak). Conducting qualitative research with CHWs regarding their motivations, perceptions, experience, and concerns about working during an infectious disease outbreak could inform larger policy decisions.

Given that a majority of emerging infectious diseases are zoonotic in origin (29), and acknowledging CHWs' competence in communicating important health concepts in a sensitive and culturally appropriate fashion, CHWs could also be used to develop and promote One Health messaging campaigns. Doing so could improve inherent resilience by leading to more successful behavioral change campaigns and increased awareness of the risks posed by environmental intrusion, bushmeat consumption, and other factors that promote infectious disease spillover events.

Third, CHWs could be used to promote adaptive resilience by serving as a type of reserve heath corps during public health emergencies. As detailed earlier, this role should be not medical which could expose CHWs to unnecessary risks, but one rooted in social mobilization. Engaging CHWs in national risk communication strategies and plans could act to simultaneously expand the reach of communication networks and enhance the perceived validity of the information dispersed by them. This could help to reduce the risk of misinformation and rumors that can lead to fear, social unrest, and violence during an outbreak response.

Finally, studies have shown that the late engagement of CHWs can hinder an outbreak response (30). Thus, formalizing or developing agreements that quickly engage CHWs during public health emergencies could improve overall response procedures and improve adaptive resilience. At a minimum, the experience, local-level knowledge, and relationships of CHWs could help to inform and guide higher-level efforts, but clearly defining CHW roles and expectations in an outbreak response would bolster response activities. This is especially true in less-permissive environments, where high levels of mistrust are common and CHWs social standing can provide them with some level of protection. Indeed, CHW programs in the Central African Republic demonstrated that they could continue some level of care at all times, reach those most vulnerable populations, and maintain disease surveillance activities even in conflict zones (31).

## Conclusions

The guidance outlined in the IHR and JEE Tool provides a framework to promote global health security and pandemic preparedness where capacities already exist. However, access to these capacities is not always a reality, and CHWs represent a proven strategy for improving access to healthcare. Through their routine work, CHWs contribute to inherent resilience and pandemic preparedness by increasing access to health products and services, distributing health information, and reducing the burden felt by the formal healthcare system—all of which act to buffer against emergencies. CHWs can also contribute to adaptive resilience by increasing social mobilization, completing surveillance activities, and by filling health systems gaps left in the wake of infectious disease outbreaks. Recognizing that CHWs already play a role in pandemic preparedness, the roles and responsibilities of CHWs in pandemic preparedness could be expanded to improve health security and community-level resilience.

## Author Contributions

MB contributed to the conception and design of the manuscript and wrote the first draft of the manuscript. RK made substantial contributions to the conception of the manuscript. Both of the authors contributed to manuscript revision, and have read and approved the submitted version.

### Conflict of Interest Statement

The authors declare that the research was conducted in the absence of any commercial or financial relationships that could be construed as a potential conflict of interest.
